# Targeting the Hippo/YAP-TAZ pathway increases X-ray sensitivity in chondrosarcoma spheroids and is associated with autophagic disruption

**DOI:** 10.1016/j.jbo.2026.100785

**Published:** 2026-07-13

**Authors:** Birgit Lohberger, Dietmar Glänzer, Heike Kaltenegger, Dagmar Kolb

**Affiliations:** a Department of Orthopedics and Trauma, Medical University of Graz, Auenbruggerplatz 5, 8036 Graz, Austria; b Core Facility Ultrastructural Analysis, Medical University of Graz, Stiftingtalstrasse 24/1, 8010 Graz, Austria; c Gottfried Schatz Research Center, Cell Biology, Histology and Embryology, Medical University of Graz, Neue Stiftingtalstrasse 6, 8010 Graz, Austria

**Keywords:** Chondrosarcoma, Verteporfin, X-ray irradiation, Hippo YAP/TAZ pathway

## Abstract

**Background:**

Chondrosarcomas are malignant cartilage-forming bone tumors with limited therapeutic options, as surgical resection remains the only curative treatment, while chemo- and radiotherapy show limited efficacy due to intrinsic resistance mechanisms. Dysregulation of the Hippo signaling pathway been associated with tumor progression and therapy resistance and has emerged as a promising experimental target across several cancer entities. However, its relevance in chondrosarcoma remains unclear, particularly with regard to potential radiosensitizing effects.

**Methods:**

Human chondrosarcoma (SW-1353 and Cal78) and healthy chondrocyte (HC) spheroids were treated with Verteporfin (VP) alone or in combination with X-ray irradiation. Cell viability, Hippo/YAP-TAZ signaling, autophagy-associated markers, DNA damage, apoptosis, ultrastructural alterations, and gene expression were analyzed using ATP assays, immunoblotting, RT-qPCR, immunohistochemistry, and transmission electron microscopy.

**Results:**

VP treatment caused a dose-dependent decline in cell proliferation and suppressed key YAP/TAZ target genes, both in chondrosarcoma cells and HC, alongside downregulation of Hippo pathway components and proliferative markers like cMyc and PCNA. Combined with X-ray irradiation, these effects were amplified, supporting a potential radiosensitizing activity of VP in this preclinical model. Ultrastructural and molecular data showed that VP disrupted autophagic balance in all cell types, leading to mitochondrial damage, lipid accumulation, and nuclear fragmentation. Despite the higher basal autophagy in HC cells, VP led to cell death involving autophagy, apoptosis, and DNA damage, evidenced by increased LC3B-II, Beclin, γH2AX, and PARP cleavage. Enhanced DNA damage markers and altered p53-MDM2 signaling after treatment reflect the genotoxic stress induced.

**Conclusion:**

Our data suggest that VP, particularly in combination with irradiation, is associated with disruption of multiple survival pathways in chondrosarcoma cells, offering a promising avenue for therapeutic intervention. Future studies should investigate the interplay between autophagy, apoptosis, and Hippo signaling to enable their therapeutic targeting in cancer.

## Highlights

• VP inhibits YAP/TAZ and reduces chondrosarcoma cell proliferation.
• VP enhances X-ray response in chondrosarcoma spheroids.
• Combined VP + irradiation amplifies DNA damage and apoptosis.
• VP disrupts autophagy and induces cellular stress pathways.
• Targeting Hippo signaling is a promising therapeutic strategy.

## 1 Introduction

Treatment options for patients with chondrosarcoma are limited. Complete surgical resection remains the gold standard for managing both primary and recurrent cases, whereas radiation therapy and chemotherapy are considered supplementary treatment approaches [1], [2]. In clinical practice, Enneking-appropriate en bloc resections are not feasible for all patients due to factors such as the anatomical location of the tumor (e.g., skull base or sacrum) and the extent of disease involvement [3]. However, chondrosarcoma exhibits limited responsiveness to both chemo- and radiotherapy. This resistance is largely attributed to factors such as poor vascularization, a low proliferative rate, and the presence of a dense hyaline cartilage matrix that hinders drug and radiation access to tumor cells. Moreover, overall survival and prognosis are influenced by the histological grade and tumor subtype within this heterogeneous group of locally aggressive and malignant bone tumors [4]. Due to the poor radiosensitivity of chondrosarcoma, high radiation doses are recommended in palliative care settings, following incomplete resections, or for unresectable tumors located in anatomically challenging regions. There is broad agreement on the need for developing novel therapeutic approaches to complement surgical resection and radiation therapy. Targeted therapies aimed at specific molecular pathways have shown promising potential compared to conventional chemotherapy [5].

One of these promising signaling pathways is the Hippo signaling, which is a highly conserved regulatory network that plays a crucial role in controlling organ size, cell proliferation, apoptosis, and stem cell self-renewal [6]. Dysregulation of Hippo signaling, particularly overactivation of YAP/TAZ, is linked to tumorigenesis, metastasis, and therapy resistance in various cancers, including primary bone tumors [7]. Inhibiting YAP/TAZ activity, for instance, has shown potential in for various types of tumors such as gastric cancer [8], cervical cancer [9], or breast cancer [10] as single therapy or as combination therapy with Metformin [11]. In vitro studies have already been conducted to evaluate the efficacy of Verteporfin (VP) in the context of malignant bone tumors such as osteosarcoma [12], [13] or Ewing sarcoma [14]. VP is an FDA-approved photosensitizer used to treat neovascular macular degeneration [15]. During photodynamic therapy, VP requires activation by nonthermal red light to generate cytotoxic oxygen species, resulting in localized cell and tissue damage. Beyond its light-activated function, VP is also widely used in experimental research to inhibit the interaction between YAP and TEAD.

Currently, no data is available regarding regarding the efficacy of VP in chondrosarcoma. One objective of this study was to explore the anticancer effects of VP, a drug that interferes with the YAP-TEAD interaction, on human chondrosarcoma cells, extending its application beyond its original role in photodynamic therapy [16]. Additionally, the impact on potential radiosensitization of the cells was examined. To achieve the most physiological response, 3D spheroids were utilized, allowing the cells to communicate intercellularly and organize themselves in a manner similar to natural tissue. These characteristics enhance the accuracy of predicting treatment outcomes. Our previous studies demonstrated that both X-ray and particle irradiation (IR) resulted in reduced chondrosarcoma cell survival, a G_2_/M phase cell cycle arrest, and a reprogramming of cellular metabolism. However, the majority of the clearly visible DNA damage caused by IR was regenerated within 24 h, and the metabolic phenotype was subsequently restored [17], [18]. Therefore, inhibiting these highly efficient DNA repair mechanisms represents a logical next step in enhancing radiosensitivity and potentially improving the effectiveness of radiotherapy.

## 2 Methods

### 2.1 3D spheroid cultures

SW-1353 (ATCC® HTB-94™, LGC Standards, Middlesex, UK) and Cal78 (ACC449; DSMZ, Leibniz, Germany) chondrosarcoma cell lines were cultured in Dulbecco's-modified Eagle's medium (DMEM-HG) supplemented with 10% FBS, 1% l-glutamine, 1% penicillin/streptomycin, and 0.1% amphotericin B (all GIBCO®, Invitrogen, Darmstadt, Germany). The cell lines were verified by short tandem repeat analysis utilizing PowerPlex™ 16 System Kit (Promega, Mannheim, Germany). Healthy human chondrocytes (HC), isolated from a 65-year-old Caucasian male and obtained from Cell Application, Inc. (San Diego, CA, USA), were utilized as control cells up to passage 4. To prevent and mitigate chondrocyte dedifferentiation, a chondrocyte-specific growth medium was employed in all experiments. This medium consisted of DMEM/F12 supplemented with 10% fetal bovine serum (FBS), 1% Penicillin-Streptomycin (5,000 U/mL), 1% l-Glutamine, 0.1% amphotericin B, 1% Insulin-Transferrin-Selenium, 0.01% TGF-β (1 ng/mL), and 0.01% FGF (1 ng/mL), all sourced from Gibco Invitrogen. All experiments were performed with mycoplasma-free cells. A total of 2,000 cells per spheroid were seeded onto the MicroTissues® 3D Petri Dish® micro-molds (Merck, Darmstadt, Germany). Cell-to-cell adhesion facilitated within 48 h the self-assembly of 3D spheroids. For each group, 256 spheroids were prepared in triplicate. Before RNA and protein extraction, the 3D spheroids were mechanically dissociated by repeated pipetting.

### 2.2 Cell viability assay

Cell viability were analyzed using the CellTiter-Glo® 3D Cell Viability Assay (Promega) according to the manufacturer's recommendations. The luminescence-based assay quantifies ATP, a marker of metabolically active cells, providing a uniform approach to measuring the viability of cells in 3D cultures. 3D spheroids were treated with 0–25 μM Verteporfin (VP; Selleckchem, Houston, TX, USA) and analyzed after 48 h (*n* = 6; measured in triplicates).

### 2.3 X-ray irradiation set up

X-ray irradiation (IR) was performed at the Division of Biomedical Research, Medical University Graz, with a RS-2000 biological irradiator (RadSource Technologies Inc., Buford, GA, USA) equipped with a 3 mm Al / 0.3 mm Cu filter and a current of 25 mA. This set-up provides 160 kV X-rays at a dose rate of 2.108 Gy/min.

### 2.4 Electron microscopy

1 h and 48 h after treatment with 5 μM VP, 8 Gy X-ray IR, or a combined treatment with 5 μM VP + 8 Gy X-ray IR, the 3D spheroids were fixed in 2.5% (wt/vol) glutaraldehyde and 2% (wt/vol) paraformaldehyde in 0.1 M cacodylate buffer (pH 7.4) for 2 h on Aclar film (Gröpl, Tulln, Austria). The samples were post-fixed with 2% (wt/vol) osmium tetroxide for 2 h at room temperature, dehydrated through a graded ethanol series, and embedded in TAAB epoxy resin (Agar Scientific, Essex, UK). Ultrathin sections (70 nm thick) were prepared using a UC 7 ultramicrotome (Leica Microsystems, Vienna, Austria), then stained with lead citrate for 5 min followed by platinum blue for 15 min. Imaging was performed with a Tecnai G2 20 transmission electron microscope (Thermo Fisher), equipped with a Gatan Ultrascan 1000 CCD camera (operating at −20 °C) and Digital Micrograph acquisition software (Gatan, Munich, Germany), at an acceleration voltage of 120 kV.

### 2.5 Protein expression analysis

Whole cell protein extracts were prepared with lysis buffer (50 mM Tris-HCl pH 7.4, 150 mM NaCl, 1 mM NaF, 1 mM EDTA, 1% NP-40, 1 mM Na3VO4) and a protease and phosphatase inhibitor cocktail (Sigma Aldrich), after 1 h and 48 h after VP treatment, respectively 8 Gy X-ray IR. The following groups have been isolated: Untreated control and 2.5, 5, and 10 μM VP treatment; as well as untreated non-IR controls, 5 μM VP treated non-IR, 8 Gy X-ray IR, and the combined treatment of 5 μM VP and 8 Gy X-ray IR samples. Protein concentration was determined with the Pierce BCA Protein Assay Kit (Thermo Fisher Scientific). The proteins were separated by SDS-PAGE and were blotted on Amersham™ Protran™ Premium 0.45 μM nitrocellulose membranes (GE Healthcare Life Science, Little Chalfont, UK). Primary antibodies against the Hippo signaling components MST1/2, SAV1, MOB/p-MOB, LATS1, YAP/TAZ/p-YAP(Ser127)/p-YAP(Ser397); the YAP/TAZ transcriptional targets CTGF, CYR61, Integrin β2, Axl, IGFBP3, Lamin B2; the autophagy marker LC3BI-II; the apoptosis markers cleaved caspase 3 and cleaved PARP; and the DNA damage marker phospho-histone γH2AX (all Cell Signaling Technology, Danvers, MA) and β-actin as loading control were used. For LC3B I-II immunoblotting cells were treated with the lysosomal protease inhibitors E64d and pepstatin A (10 μg/mL; both Sigma Aldrich), which were used to block autophagic flux and inhibit the degradation of LC3B-II [19]. Blots were developed using a horseradish peroxidase-conjugated secondary antibody (Dako) for 1 h and the Amersham™ ECL™ prime western blotting detection reagent (GE Healthcare). Chemiluminescence signals were detected with the ChemiDocTouch Imaging System (BioRad Laboratories Inc., Hercules, CA) and images were processed with the ImageLab 5.2 Software (BioRad Laboratories Inc).

### 2.6 Quantitative PCR (qPCR)

Total RNA was isolated 48 h after treatment using the RNeasy Mini Kit and DNase-I treatment according to the manufacturer's manual (Qiagen, Hilden, Germany). The following groups have been isolated: untreated non-IR controls, 5 μM VP treated non-IR, 8 Gy X-ray IR, and the combined treatment group consisting of 5 μM VP and 8 Gy X-ray IR samples. Two μg RNA were reverse transcribed with the iScript-cDNA Synthesis Kit (BioRad Laboratories Inc.) using a blend of oligo(dT) and hexamer random primers. Amplification was performed with the SsoAdvanced Universal SYBR Green Supermix (Bio-Rad Laboratories Inc.) using technical triplicates and measured by the CFX384 (BioRad Laboratories Inc.). The following QuantiTect primer assays (Qiagen) were used for real time RT-PCR: *BIRC5*, *cMyc*, *PCNA* (proliferation); *BECN* (autophagy); *GADD45A*, *MDM2*, and *TP53* (DNA damage). Results were analyzed using the CFX manager software for CFX Real-Time PCR Instruments (Bio-Rad Laboratories Inc., version 3.1) software and quantification cycle values were exported for statistical analysis. Results with Ct values greater than 30 were excluded from analysis. Relative quantification of expression levels was obtained by the ∆∆Ct method based on the geometric mean of the internal controls ribosomal protein, large, P0 (*RPL*) and TATA box binding protein (*TBP*), respectively. Expression level (C_t_) of the target gene was normalized to the reference genes (ΔC_t_), the ΔC_t_ of the test sample was normalized to the ΔC_t_ of the control (ΔΔC_t_). Finally, the expression ratio was calculated with the 2^-ΔΔCt^ method (*n* = 6; biological triplicates).

### 2.7 γH2AX immunohistochemical staining

3D spheroid cultures of controls, 5 μM VP, 8 Gy X-ray IR, and combined 5 μM VP + 8 Gy X-ray IR were paraffin-fixed 48 h after treatment. Following deparaffinization and rehydration, sections underwent microwave-assisted antigen retrieval in 10 mM sodium citrate buffer (pH 6.0) containing 0.05% Tween-20 (Thermo Fisher Scientific). Endogenous peroxidase activity was quenched with 1% hydrogen peroxide (Merck, Darmstadt, Germany) at room temperature for 10 min. Sections were then incubated with the primary anti-γH2AX antibody (1:200; Merck) at room temperature for 1 h. For primary antibodies of rodent origin, a bridging step using the primary antibody enhancer (1:100; Dako, Glostrup, Denmark) was performed prior to HRP-conjugated secondary antibody treatment. Positive staining was developed with AEC (Abcam, Cambridge, UK), and nuclei were counterstained with hematoxylin.

### 2.8 Statistical analysis

Statistical analyses were conducted using IBM SPSS Statistics 29.0.0.0 (241) (New York, NY, USA), while graphical representations were generated with SigmaPlot 14.5 (SYSTAT, Palo Alto, CA, USA). Given the limited sample size, a conservative statistical approach was adopted, and non-parametric methods were applied throughout the study to avoid assumptions regarding data distribution. The Mann–Whitney *U* test was used for pairwise comparisons, while the Kruskal–Wallis H test, followed by Bonferroni-corrected pairwise comparisons, was applied for multiple-group analyses. *P*-values were considered statistically significant at thresholds of <0.05 */#, <0.01 **/##, and ***<*** *0.001* ***/###.

## 3 Results

### 3.1 Verteporfin inhibited the Hippo pathway and their corresponding transcriptional targets dose dependently in chondrosarcoma cells

**Fig. 1:**
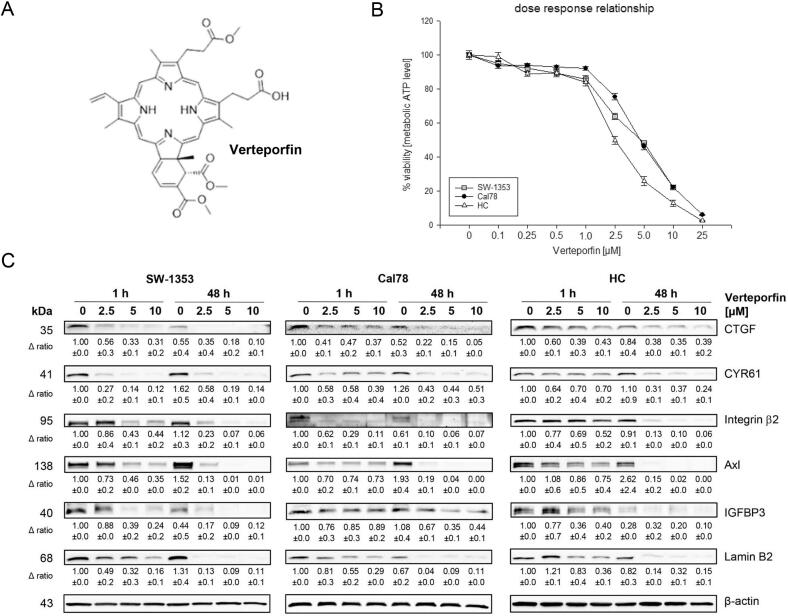
Effect of Verteporfin on chondrosarcoma cells and healthy chondrocytes. A) Chemical structure of Verteporfin (VP); B) Cell growth of SW-1353 and Cal78 chondrosarcoma cells, as well as healthy chondrocytes (HC), was inhibited in a dose-dependent manner (mean ± SD, n = 6, measured in biological triplicates) 48 h after treatment. C) Protein expression of the YAP/TAZ transcriptional targets CTGF, CYR61, Integrin β2, Axl, IGFBP3, and Lamin B was analyzed by immunoblotting under control conditions versus after treatment with 2.5, 5, and 10 μM VP for 1 h and 48 h. β-actin was used as loading control. Δ ratio, fold change was normalized to non-treated controls (mean ± SD of n = 3). Full-length blots are presented in supplementary Fig. S1.

To investigate the cytotoxic effects of Verteporfin (VP; Fig. 1A) on human chondrosarcoma cell lines, respectively human healthy chondrocytes (HC) as comparison group, cells were treated with 0–25 μM VP, and the dose-response relationship was assessed after 48 h. Both chondrosarcoma cells and HC exhibited a dose-dependent reduction in cell viability following VP treatment (Fig. 1B). The most pronounced effects, indicated by the lowest IC_50_ values, were observed in HC (IC_50_ 2.6 ± 0.2 μM for HC; 5.8 ± 0.8 μM for SW-1353; 4.9 ± 0.2 μM for Cal78; mean ± SD of *n* = 6, measured in triplicates). Protein expression and phosphorylation analysis were examined through immunoblotting and revealed a dose dependent inhibition of the YAP/TAZ transcriptional targets CTGF, CYR61, Integrin β2, Axl, IGFBP3, and Lamin B after treatment with 2.5, 5, and 10 μM VP for 1 h and 48 h (Fig. 1C). For the majority of targets, SW-1353 cells showed the most sensitive response 1 h after treatment, while the inhibitory effect on the HC occurred only after an extended duration of treatment time. The relative changes of protein expression or phosphorylation, presented as fold change (Δ-ratio), were normalized to the untreated control group. β-actin was used as loading control (mean ± SD of *n* = 3).

**Fig. 2:**
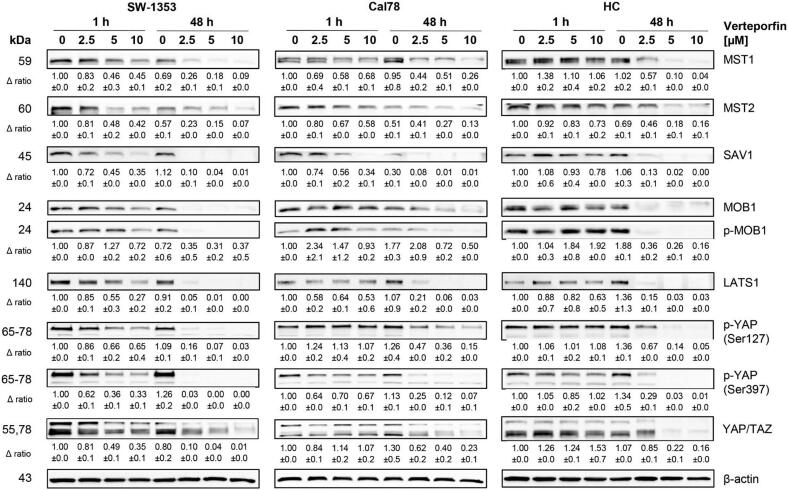
Inhibition of Hippo YAP/TAZ signaling through Verteporfin. Protein expression and phosphorylation of the Hippo signaling cascade MST1, MST2, SAV1, LATS1, YAP/TAZ, as well as YAP(Ser127) and YAP(Ser397) were analyzed by immunoblotting under control conditions versus after treatment with 2.5, 5, and 10 μM VP for 1 h and 48 h. β-actin was used as loading control. Δ ratio, fold change was normalized to non-treated controls (mean ± SD of n = 3). Full-length blots are presented in supplementary Fig. S1.

Protein expression and phosphorylation of the Hippo YAP/TAZ signaling pathway components showed a similar progression (Fig. 2). Quantification of the bands revealed that MST1, MST2, SAV1, LATS1, YAP/TAZ expression, respectively phosphorylation of YAP(Ser127) and YAP(Ser397) was 50% lower in 5 μM treated samples compared to control after 1 h in SW-1353 cells. Cal78 cells exhibited a similar response, but YAP(Ser127) and YAP(Ser397) were not affected at this time point. Likewise, healthy HC showed no effect 1 h after treatment, with expression or phosphorylation inhibition occurring only after 48 h.

### 3.2 Ultrastructural alterations following combined Verteporfin and X-ray irradiation treatment

**Fig. 3:**
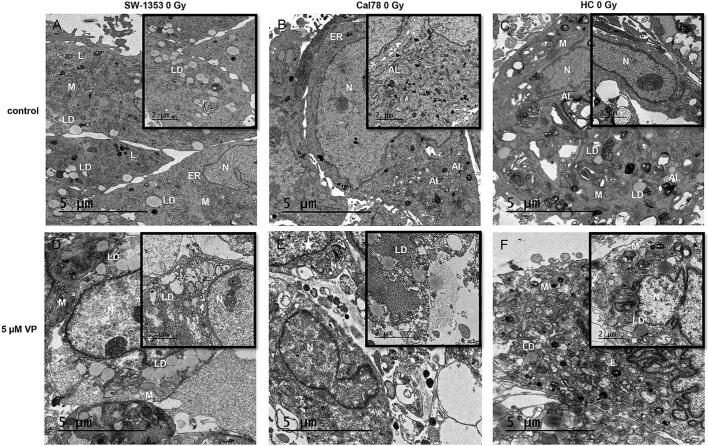
Ultrastructure analysis of 3D spheroid cultures after VP treatment using transmission electron microscopy. A) SW-1353, B) Cal78, and C) HC spheroid cultures were analyzed under control conditions and D/E/F) after 5 μM VP treatment for 48 h. The following abbreviations have been used: Lipid droplets (LD), nucleus (N), small dark lysosomes (L), endoplasmic reticulum (ER), mitochondria (M), autophagolysosomes (AL), cell shrinkage - lipid droplets (LD), bright ultrastructure of heterochromatin (asterisk).

The ultrastructure of cells in 3D spheroid cultures was analyzed using transmission electron microscopy. SW-1353 control cells displayed a high abundance of lipid droplets (LDs), a normal morphology, and an intact nuclear ultrastructure. Additionally, the cells contained small dark lysosomes (L), an endoplasmic reticulum (ER), and mitochondria (M), all of which appeared unaffected (Fig. 3A). Cal78 cells under control conditions displayed autolysosomes (AL) and a normal nuclear shape (N). The tubular structures of the endoplasmic reticulum (ER) and the mitochondrial (M) ultrastructure appeared unremarkable (Fig. 3B). HC control cells showed a substantial accumulation of autophagolysosomes (AL). The nucleus (N), lipid droplets (LDs), endoplasmic reticulum (ER), and mitochondria (M) maintained their typical morphology (Fig. 3C).

Treatment with 5 μM VP induced structural alterations in SW-1353 cells, including lipid remnants surrounding the nucleus. The mitochondrial (M) ultrastructure appeared compromised, showing minimal signs of normal functionality (Fig. 3D). Cal78 cells treated with 5 μM VP displayed signs of cellular damage, including nuclear (N) shrinkage and fragmentation, a bright ultrastructure of heterochromatin (asterisk), and a cytoplasm lacking clear structural organization. Remnants of lipid droplets (LD) were also observed (Fig. 3E). In contrast, HC cells treated with 5 μM VP showed nuclear fragmentation, a bright ultrastructure of heterochromatin, and a disorganized cytoplasm, with a compact distribution of small lysosomes (L). The mitochondrial (M) ultrastructure was disturbed, and lipid droplets (LD) were present (Fig. 3F).

**Fig. 4:**
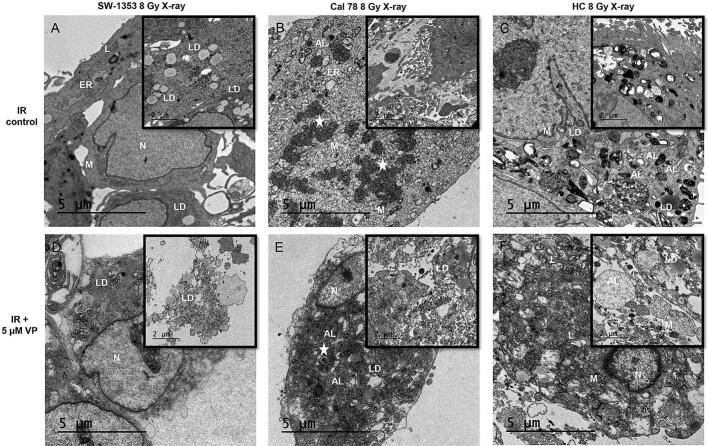
Ultrastructure analysis of 3D spheroid cultures after combined treatment with VP and X-ray irradiation (IR) using transmission electron microscopy. A) SW-1353, B) Cal78, and C) HC cells were analyzed under X-ray IR and D/E/F) after the combined treatment with 5 μM VP treatment and 8 Gy X-ray IR for 48 h. The following abbreviations have been used: Lipid droplets (LD), nucleus (N), small dark lysosomes (L), endoplasmic reticulum (ER), mitochondria (M), autophagolysosomes (AL), cell shrinkage - lipid droplets (LD), bright ultrastructure of heterochromatin (asterisk).

Under the influence of X-ray irradiation SW-1353 cells showed high amount of lipid droplets (LD), ultrastructural changes of the nucleus (N), some small dark lysosomes (L), dilated endoplasmic reticulum (ER), whereas mitochondria (M) were unaffected (Fig. 4A). Irradiated Cal78 cells showed a high amount of autophagolysosomes (AL), small fragments of endoplasmic reticulum (ER) and affected mitochondria. Dark unspecific areas within the cytoplasm (asterisks) were distinguishable (Fig. 4B). Additionally, after irradiation with 8 Gy X-ray, the healthy HC cells exhibited a high abundance of autophagolysosomes (AL), a fragmented nuclear shape (N), dark lipid droplets (LDs), and dilation of the endoplasmic reticulum (ER) and mitochondria (M) (Fig. 4C). An additional treatment with 5 μM VP resulted in significant shrinkage of SW-1353 cells, leaving only remnants of lipid droplets (LDs). The nucleus (N) appears notably large in comparison to the surrounding cytoplasm (Fig. 4D). The Cal78 chondrosarcoma cells exhibited severe ultrastructural damage following the combined treatment. Bright regions are visible within the nucleus (N), while the cytoplasm appears disorganized, with compact dark areas and remnants of lipid droplets (LD) present (Fig. 4E). In HC cells, the nucleus (N) appeared small and shrunken, with heterochromatin exhibiting a light-colored ultrastructure. The cytoplasm was densely packed and lacked clear organization. A large number of small, nonspecific lysosomes (L) and autophagolysosomes (AL) were present, while the mitochondrial (M) ultrastructure was disrupted. Remnants of lipid droplets (LD) were also observed (Fig. 4F).

### 3.3 Effects of combined Verteporfin and X-ray irradiation on autophagy and DNA damage in chondrosarcoma cells

**Fig. 5:**
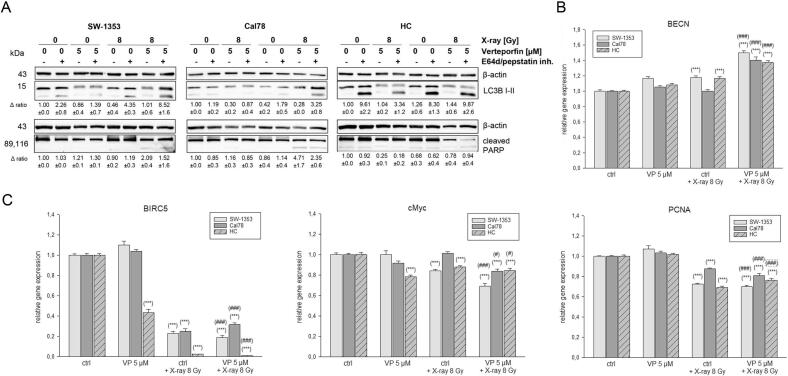
Impact of combined treatment on autophagy and survival. A) Western blot analysis of whole 3D spheroid lysates to assess autophagy marker expression in the chondrosarcoma cell lines SW-1353, Cal-78, and human healthy chondrocytes (HC) treated with 5 μM VP alone or with combined treatment with 8 Gy X-ray IR for 48 h. Each group was analyzed in the presence (+) or absence (−) of the lysosomal protease inhibitors E64d and pepstatin A (Inh), which block autophagic flux by preventing lysosomal degradation of LC3B-II, resulting in its accumulation.Relative gene expression of B) the autophagy marker Beclin (BECN) and C) the proliferation and cell survival markers BIRC5, cMyc, and PCNA (mean ± SD, n = 6, measured in triplicates). Statistical significances to the untreated controls are defined as follows: *** p < 0.001; respectively between the 5 μM VP group and the combined treatment with IR are presented as ^#^ p < 0.05; ^###^ p < 0.001.

The enhanced autophagy observed in the electron microscopy following VP treatment and X-ray IR was also evident at the protein and RNA levels (Fig. 5A). To block the autophagic flux and prevent the degradation of LC3B-II, cells were pre-treated with the lysosomal protease inhibitors E64d and pepstatin A. Both chondrosarcoma cell lines exhibited a significant increase in LC3B-II following combined treatment with 5 μM VP and 8 Gy X-ray irradiation, consistent with alterations autophagy-associated processes. Beclin (*BECN*), an upstream regulator in the autophagy cascade, showed a highly significant increase in relative gene expression following the combined treatment (SW-1353: 1.50 ± 0.1***; Cal78: 1.40 ± 0.2***; HC: 1.37 ± 0.1***) compared to both the untreated control and the VP-treated group (Fig. 5B). Significant differences between the 5 μM VP group and the combined treatment are indicated by “#”. Furthermore, a slight induction of apoptosis was observed, as evidenced by PARP cleavage in the chondrosarcoma cells. The relative changes in protein expression were presented as fold change (Δ-ratio), with β-actin used as the loading control (mean ± SD of *n* = 3).

To overcome resistance to radiotherapy in human chondrosarcoma cells, we investigated the efficacy of inhibition of cell proliferation and the effects on DNA damage response with VP in combination with X-ray IR. In order to be able to show the regulation of the important proliferation key genes *cMyc* and *PCNA*, as well as the cell survival marker *BIRC5*, after combined treatment, we performed RT-qPCR analysis with RNAs isolated 48 h after 5 μM VP ± 8 Gy X-ray (Fig. 5C). *BIRC5*, a key gene involved in cell proliferation and resistance to ionizing radiation, was highly significantly downregulated (SW-1353: 0.19 ± 0.1***; Cal78: 0.32 ± 0.1***; HC: 0.01 ± 0.0***). IR with 8 Gy X-ray as well as the combined treatment led to a significant increase in the expression of *cMyc* (SW-1353: 0.69 ± 0.1***; Cal78: 0.84 ± 0.1***; HC: 0.85 ± 0.1***) and *PCNA* (SW-1353: 0.71 ± 0.1***; Cal78: 0.80 ± 0.1***; HC: 0.75 ± 0.1***).

**Fig. 6:**
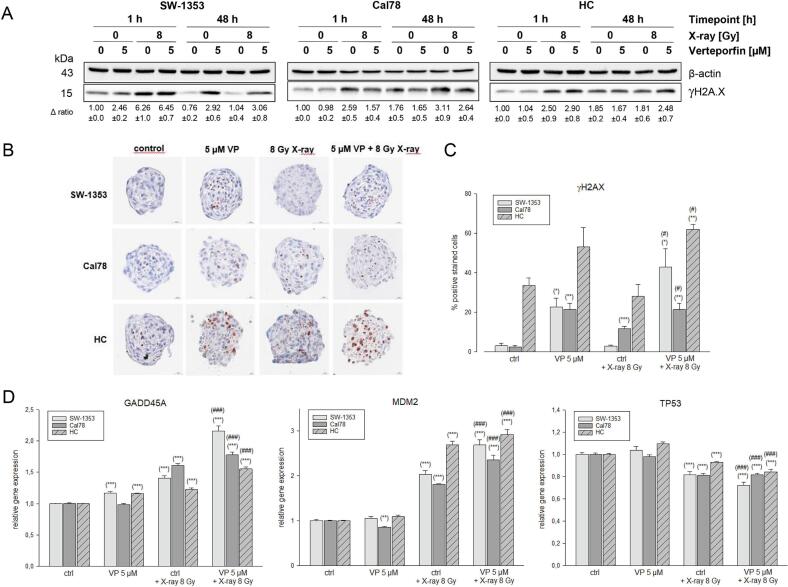
Association of VP treatment with enhanced DNA damage following irradiation. A) Protein phosphorylation of the DNA damage marker γH2AX was analyzed by immunoblotting under control conditions versus after treatment with 5 μM VP, respectively combined treatment for 48 h. β-actin was used as loading control. Δ ratio, fold change was normalized to non-treated controls (mean ± SD of n = 3). Full-length blots are presented in supplementary Fig. S1. B) Representative pictures of the γH2AX immunohistochemical staining of 3D spheroid cultures and C) the corresponding quantification using the QuPath-0.5.1 software. D) SW-1353 (light grey) and Cal78 (dark grey) chondrosarcoma cells and HC (dashed) were treated with 5 μM VP alone respectively in a combined treatment with 8 Gy X-ray IR (mean ± SD, n = 6, measured in triplicates). Relative gene expression of the DNA damage markers GADD45A, MDM2, and TP53 were analyzed 48 h after IR (mean ± SD, n = 6, measured in triplicates). Statistical significances to the untreated controls are defined as *** p < 0.001 and significances between the 5 μM VP group and the combined treatment with IR are presented as ^###^ p < 0.001.

To assess the initiation and maintenance of DNA damage, the expression of the DNA damage marker γH2AX was evaluated through protein phosphorylation (Fig. 6A) and immunohistochemistry (Fig. 6B/C). The combined treatment with VP and X-ray IR kept the cells in a state of sustained DNA damage, preventing regeneration. SW-1353 cells, in particular, are known for their rapid regeneration after IR, while the rate of γH2AX expression is generally higher in HC cells. As shown by both the representative images and the quantification, VP and the combined treatment with X-ray IR led to increased expression of γH2AX. The quantification of immunohistochemical staining in 10 spheroids per group using QuPath-0.5.1 software validated these findings. Relative gene expression of the DNA damage markers *GADD45A* (SW-1353: 2.18 ± 0.3***; Cal78: 1.78 ± 0.2***; HC: 1.55 ± 0.1***) and *MDM2* (SW-1353: 2.69 ± 0.5***; Cal78: 2.36 ± 0.4***; HC: 2.92 ± 0.5***) was significantly upregulated following the combined treatment. *TP53* expression, however, was not affected by VP treatment (SW-1353: 0.72 ± 0.1***; Cal78: 0.82 ± 0.0***; HC: 0.84 ± 0.1***). In contrast, X-ray IR and the combined treatment resulted in a significantly reduced expression (Fig. 6D).

## 4 Discussion

YAP and TAZ are transcriptional co-activators that play a central role in the Hippo signaling pathway, which regulates cell, tissue, and organ growth. Under growth conditions, YAP and TAZ translocate to the nucleus, where they interact with DNA-binding transcription factors to regulate the expression of genes involved in fundamental cell functions, such as proliferation and survival [20], [21]. VP treatment led to a dose-dependent decrease in cell proliferation in both chondrosarcoma cells and healthy chondrocytes (HC), with an IC_50_ of 5 μM in tumor cells and 2.5 μM in HC cells. The lower IC₅₀ of VP observed in HC compared with tumor cells likely reflects fundamental differences in cellular vulnerability, resulting in the greater sensitivity of healthy chondrocytes. Although this may be explained by differences in oxidative stress responses and cellular homeostasis, it also raises concerns regarding the therapeutic window of VP. Consequently, systemic application of VP may be associated with toxicity to normal cartilage, emphasizing the need for optimized dosing strategies, local drug delivery approaches, or combination regimens that maximize tumor selectivity while minimizing adverse effects on normal tissue [22]. Moreover, chondrocytes depend more strongly on intact YAP/TAZ signaling for survival and lack the stress adaptation and drug resistance mechanisms characteristic of tumor cells. Although VP exhibited higher accumulation in normal cells than in tumor cells in vivo, in vitro studies demonstrated that normal cells could efficiently clear VP-induced protein oligomers through autophagic and proteasomal pathways [23]. Together, these factors render healthy chondrocytes intrinsically more sensitive to VP exposure.

In previous work, we demonstrated that human chondrosarcoma cells have a highly efficient DNA repair program after proton irradiation within a very short period. Additionally, this process was accompanied by a reprogramming of cellular metabolism [17]. Given the complex role of Hippo signaling in various cellular processes, its potential effect on chondrosarcomas remains unclear and was therefore the focus of this study. Among the targets of this transcriptional regulation by YAP and TAZ are extracellular matrix proteins like CTGF, CYR61, and integrin β2, the inhibitor of apoptosis protein (IAP) survivin (BIRC5), the mechano-sensitive nuclear envelope protein Lamin B2, and the oncogenic receptor tyrosine kinase Axl [22]. Although chondrosarcoma cells had a higher IC_50_ value, VP more efficiently and rapidly inhibited the expression of CTGF, CYR61, integrin β2, Axl, IGFBP3, and lamin B2 with increasing concentrations compared to HC cells. The Hippo pathway is centered around a kinase cascade, with MST1/2, SAV1, LATS1/2, YAP, and TAZ serving as its key components [24]. Phosphorylation is a key mechanism regulating the Hippo pathway and controlling YAP/TAZ activity. The interactions between membrane-bound upstream regulators lead to the activation of the cytoplasmic kinases MST1/2 and LATS. The activated MST kinase binds to the adaptor protein SAV1 and phosphorylates MOB1, which in turn activates the LATS kinase. LATS then phosphorylates YAP and TAZ, leading to cytoplasmic retention or degradation, whereas reduced kinase activity allows nuclear translocation and activation of pro-proliferative genes [25]. This dynamic regulation may underlie cell type-specific differences in proliferation and survival responses. In chondrosarcoma cells, all components of this signal transduction cascade were inhibited by VP in a dose-dependent manner as early as 1 h after irradiation, whereas in HC cells, these effects became evident only after 48 h. The effectiveness of VP in regulating proliferation and migration has already been demonstrated in other sarcoma types, including rhabdomyosarcoma [26] and Ewing sarcoma [14]. However, these studies did not include healthy comparison cells. Although VP effectively inhibited Hippo/YAP-TAZ signaling and downstream target expression, the observed biological effects cannot be attributed exclusively to Hippo pathway inhibition. Given the known YAP-independent activities of Verteporfin, including mitochondrial dysfunction, ROS generation, and proteotoxic stress, multiple cellular stress pathways are likely involved. Future genetic YAP/TAZ knockdown or knockout studies will be required to distinguish YAP-dependent from YAP-independent effects.

Ultrastructural analysis of 3D spheroid cultures revealed key changes in autophagy, especially following VP treatment. As a vital process for cellular homeostasis, autophagy responds to internal conditions and external stressors like drugs [27], [28]. Our findings highlight cell-type-specific alterations in autophagy in SW-1353, Cal78, and HC cells under control and treated conditions. Under control conditions, SW-1353 cells showed intact ultrastructure with abundant lipid droplets, normal organelles (ER, mitochondria, lysosomes), and preserved nuclear architecture [29]. These features indicate stable basal autophagy and maintained cellular homeostasis without signs of stress. Cal78 cells exhibited normal nuclear morphology and tubular ER and mitochondria. However, the presence of autolysosomes (AL) suggests ongoing basal autophagy, likely reflecting routine turnover of organelles and macromolecules in response to physiological demands [30]. HC control cells showed intact ER, mitochondria, and lipid droplets, along with notable autophagolysosome (AL) accumulation. This suggests a more active autophagic process compared to SW-1353 and Cal78 cells, possibly reflecting cell-specific adaptations to growth or nutrient recycling demands in 3D spheroid cultures [31]. Treatment with 5 μM VP led to significant alterations in autophagic processes across all cell lines. In SW-1353 cells, perinuclear lipid remnants indicated impaired lipid metabolism and degradation. Mitochondrial structure was severely altered, with little sign of normal function, suggesting defective mitophagy. Together, these features point to a breakdown in the clearance of damaged organelles and lipids, typical of cellular stress or drug-induced injury [32]. In Cal78 cells, VP treatment caused pronounced nuclear changes, including shrinkage and fragmentation, indicating severe DNA damage and apoptosis. Enhanced heterochromatin brightness suggests chromatin condensation and cellular stress, potentially triggering autophagy to clear damaged nuclear components. However, disrupted cytoplasmic organization and lipid droplet remnants point to impaired autophagic flux, possibly due to defects in autophagosome formation or maturation, leading to accumulation of undegraded material. VP-treated HC cells showed nuclear fragmentation, bright heterochromatin, and cytoplasmic disorganization. The clustering of small lysosomes (L) suggests early autophagic activation, likely in response to VP-induced stress. However, mitochondrial disruption and persistent lipid droplets indicate that the autophagic response may be inadequate, with mitochondrial dysfunction potentially both triggering and worsening cellular damage [33]. Overall, these findings highlight the critical role of autophagy in maintaining cellular homeostasis in 3D spheroid cultures of SW-1353, Cal78, and HC cells. While basal autophagy is active in all cell types, with HC cells showing higher autophagolysosome accumulation under control conditions, VP treatment disrupts this balance. It leads to damaged organelles, lipid buildup, and nuclear fragmentation - hallmarks of impaired autophagy and potential cell death. The differential responses among cell lines underscore cell-type specific regulation of autophagy. Further research into the underlying mechanisms may offer insights into targeting autophagic pathways for cancer therapy.

The autophagic process was confirmed at both the protein and gene levels. The function of autophagy as an alternative mechanism of cell death has been a subject of discussion in recent years. During autophagy, the microtubule-associated protein LC3B relocates to membranes, facilitating the formation of autophagosome membranes. The level of LC3B-II serves as an indicator of autophagosome formation [19]. VP treatment, particularly in combination with X-ray irradiation, resulted in increased accumulation of LC3B-II, consistent with altered autophagy-associated processes. Furthermore, the relative expression of the Beclin gene was significantly upregulated after combined treatment. Another key indicator of potential tumor cell damage is the activation of apoptosis. Alongside autophagy, apoptosis represents a central cellular response to therapeutic interventions, determining whether tumor cells can survive or undergo programmed cell death. Monitoring apoptotic activity provides crucial insight into the efficacy of treatments, as the induction of apoptosis often correlates with reduced tumor viability and improved therapeutic outcomes. Both chondrosarcoma cell lines exhibited PARP cleavage following the combined treatment, whereas no cleavage was observed in HC cells.

*BIRC5*, also known as survivin, is a multifunctional protein with critical roles in cancer biology and interacts with and suppresses caspase-3 and caspase-7, thereby inhibiting apoptotic cell death. It may also be involved in autophagic pathways and cellular stress response mechanisms.

An siRNA screening of 51 apoptosis-related genes in chondrosarcoma cells identified *BIRC5* as crucial for chondrosarcoma cell survival [34]. A previous study has already demonstrated the significant reduction in chondrosarcoma cells caused by irradiation [35]. The oncogenic transcription factor *cMyc* serves as a key regulator of cellular growth and metabolism and has been identified as a radiosensitive locus in breast cancer [36]. Our data demonstrated a significant reduction in *cMyc* and *PCNA* expression following X-ray irradiation and the combined treatment with VP in chondrosarcoma cells.

When DNA damage occurs, a complex network of signaling cascades is triggered to help ensure the survival of the cell. γH2AX formation occurs right after DNA double-strand breaks and is widely used as a biomarker to assess DNA damage following irradiation. An increased number of γH2AX foci indicates more severe DNA damage, and these foci may remain until the damage is repaired or, if irreparable, the cell undergoes apoptosis [37]. γH2AX acts as a platform to recruit other DNA repair proteins, aiding in the localization and concentration of these repair factors at the damage sites to ensure efficient repair [38]. One hour after irradiation, a notable increase in γH2AX phosphorylation was observed in all cell lines. Immunohistochemical staining clearly revealed that the HC 3D spheroids exhibited generally higher levels of DNA damage. The addition of VP treatment further significantly elevated γH2AX levels, indicating that the DNA damage was more extensive and persisted for a longer duration. These findings are consistent with a potential radiosensitizing effect of VP. However, the present study does not establish radiosensitization as a direct consequence of Hippo pathway inhibition or autophagic disruption.

The γH2AX data is further supported by the significantly elevated expression of the DNA damage markers *GADD45A*, *MDM2*, and *TP53*. *MDM2* plays a crucial role in cellular responses to both ionizing and UV radiation. The degree to which radiation activates the p53 tumor suppressor is influenced by the level of *MDM2* expression [39]. Acute DNA damage activates p53, which triggers the expression of MDM2. This feedback loop between the two genes causes MDM2 to inhibit p53, regulating cell cycle progression and preventing excessive cell cycle arrest, thereby promoting cell survival and repair processes. Our results have shown that X-ray irradiation and, to an even greater extent, combined treatment with VP increased MDM2 expression and decreased p53 expression. Although our ultrastructural and molecular analyses consistently demonstrated alterations in autophagy-associated markers following VP treatment, these findings remain correlative. The present study was not designed to establish whether autophagic disruption directly mediates the observed radiosensitizing effect. Future studies using pharmacological or genetic modulation of autophagy will be required to determine whether altered autophagy represents a causal mechanism or a downstream consequence of VP-induced cellular stress.

## 5 Conclusion

VP modulated multiple cellular pathways in chondrosarcoma spheroids, including YAP/TAZ signaling, autophagy-associated processes, and DNA damage responses. In combination with X-ray irradiation, VP was associated with enhanced cytotoxicity and persistent DNA damage, supporting its potential radiosensitizing activity in this preclinical 3D model. However, the present data do not establish a causal relationship between autophagic disruption and radiosensitization, nor do they distinguish YAP/TAZ-dependent from YAP-independent effects of VP. Moreover, the increased sensitivity observed in healthy chondrocytes highlights the need for careful evaluation of the therapeutic window. Future studies using genetic approaches and in vivo models will be essential to define the underlying mechanisms and to evaluate the translational potential of VP for chondrosarcoma therapy.

## CRediT authorship contribution statement

**Birgit Lohberger:** Writing – original draft, Visualization, Validation, Supervision, Project administration, Methodology, Investigation, Funding acquisition, Conceptualization. **Dietmar Glänzer:** Writing – review & editing, Methodology, Investigation, Data curation. **Heike Kaltenegger:** Writing – review & editing, Methodology, Investigation, Data curation. **Dagmar Kolb:** Writing – original draft, Methodology, Investigation.

## Patient consent statement

Not applicable.

## Ethics approval statement

Not applicable.

## Funding

This work was supported by the Medical University Graz. The funding source had no role in the study design, in the collection, analysis, or interpretation of the data, in the writing of the report, or in the decision to submit the article for publication. Open access funding provided by Medical University of Graz.

## Declaration of competing interest

The authors declare the following financial interests/personal relationships which may be considered as potential competing interests: Birgit Lohberger reports financial support was provided by Medical University of Graz. If there are other authors, they declare that they have no known competing financial interests or personal relationships that could have appeared to influence the work reported in this paper. Dietmar Glaenzer reports financial support was provided by Medical University of Graz. If there are other authors, they declare that they have no known competing financial interests or personal relationships that could have appeared to influence the work reported in this paper. Heike Kaltenegger reports financial support was provided by Medical University of Graz. If there are other authors, they declare that they have no known competing financial interests or personal relationships that could have appeared to influence the work reported in this paper. Dagmar Kolb reports financial support was provided by Medical University of Graz. If there are other authors, they declare that they have no known competing financial interests or personal relationships that could have appeared to influence the work reported in this paper.

## Data Availability

All data supporting the findings of this study are included within the article.
