# Supplementary material for: Targeting the Hippo/YAP-TAZ pathway increases X-ray sensitivity in chondrosarcoma spheroids and is associated with autophagic disruption
Source: J Bone Oncol. 2026 Jul 13;59:100785. doi: 10.1016/j.jbo.2026.100785 (PMC13382328; doi:10.1016/j.jbo.2026.100785)

supplementary data
uncropped files
western blots Figure 1C+2+5A+6A

Ad Figure 1C: uncropped files

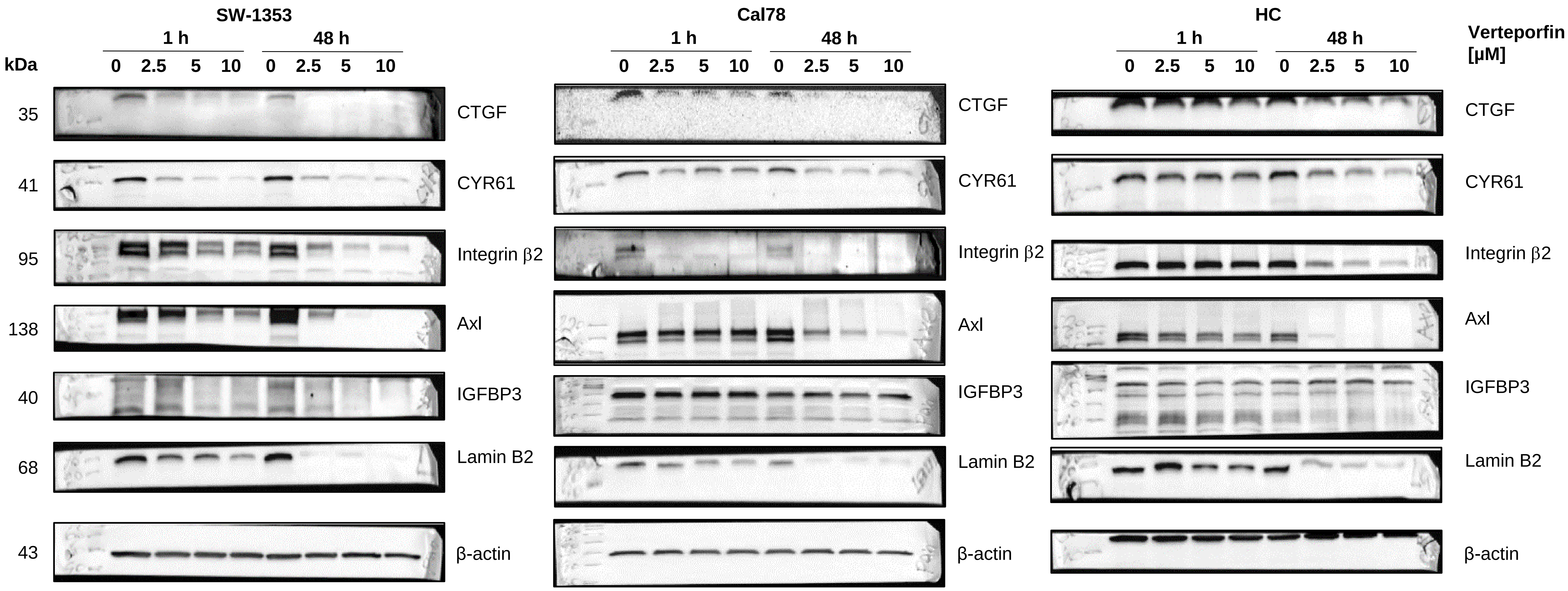

Ad Figure 2: uncropped files

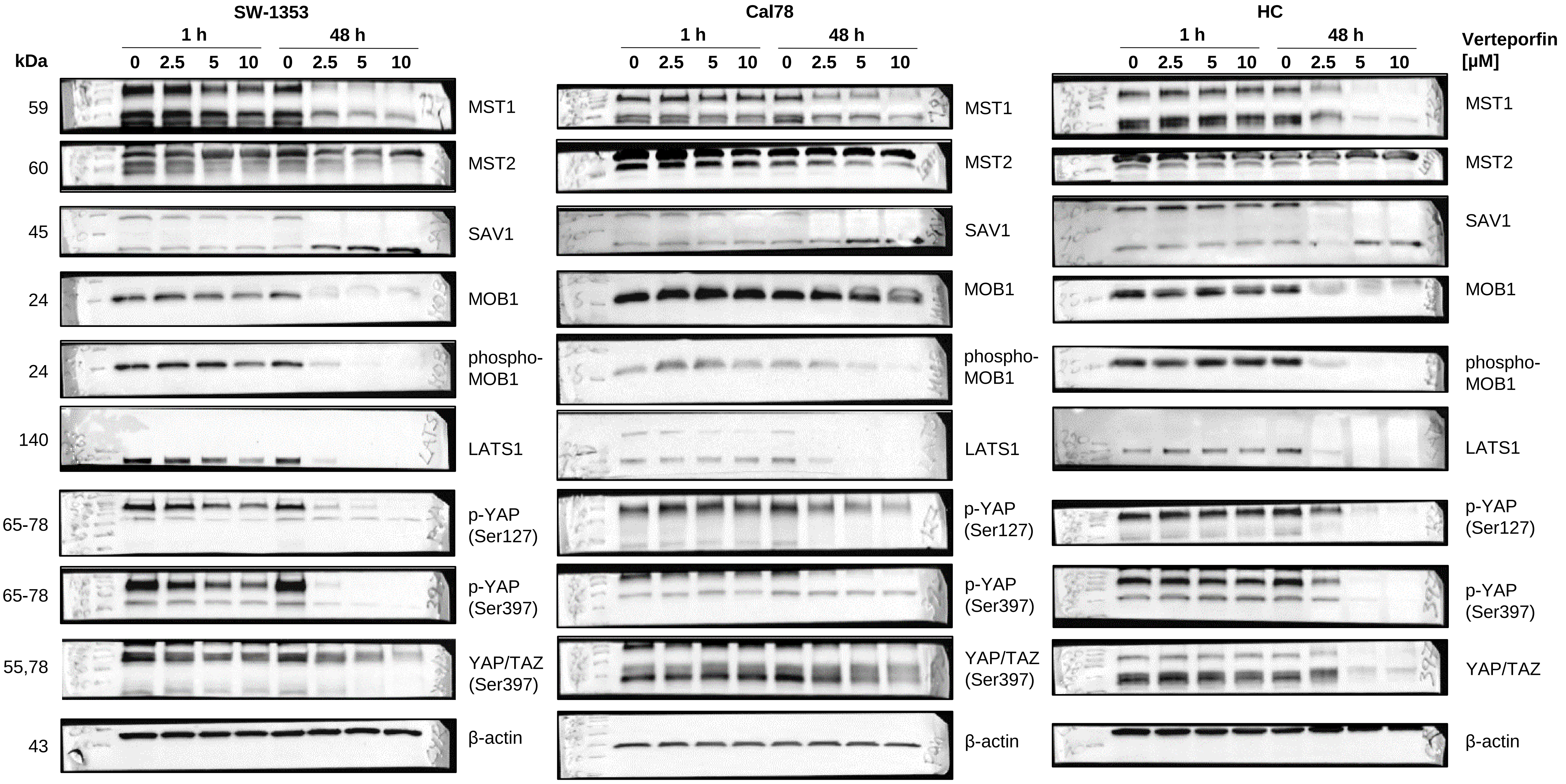

Ad Figure 5A: uncropped files (autophagy, apoptosis)

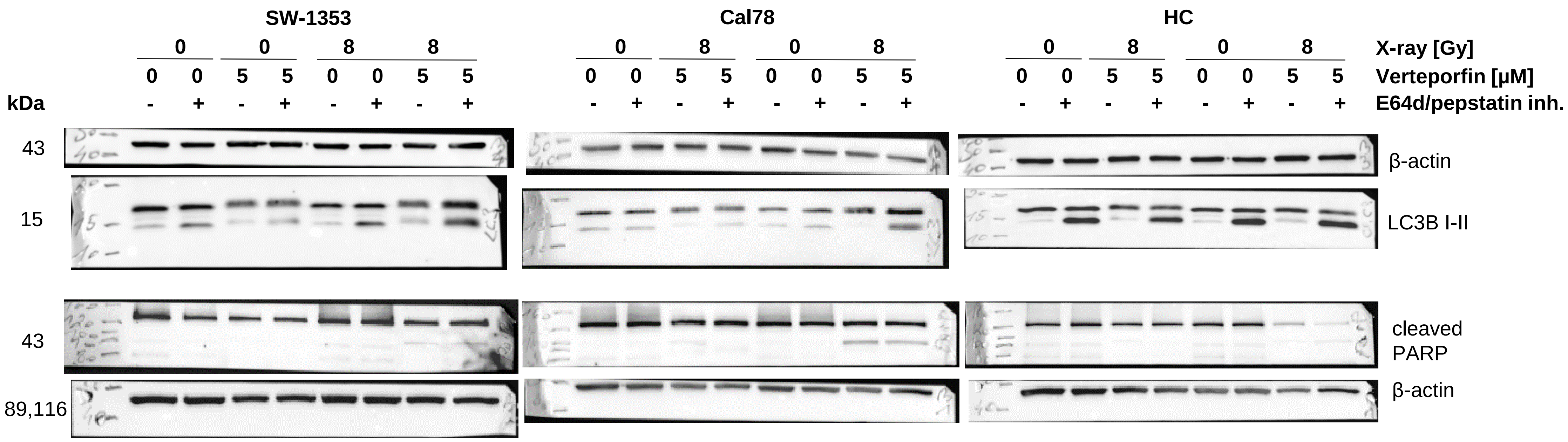

Ad Figure 6A: uncropped files (γH2A.X)

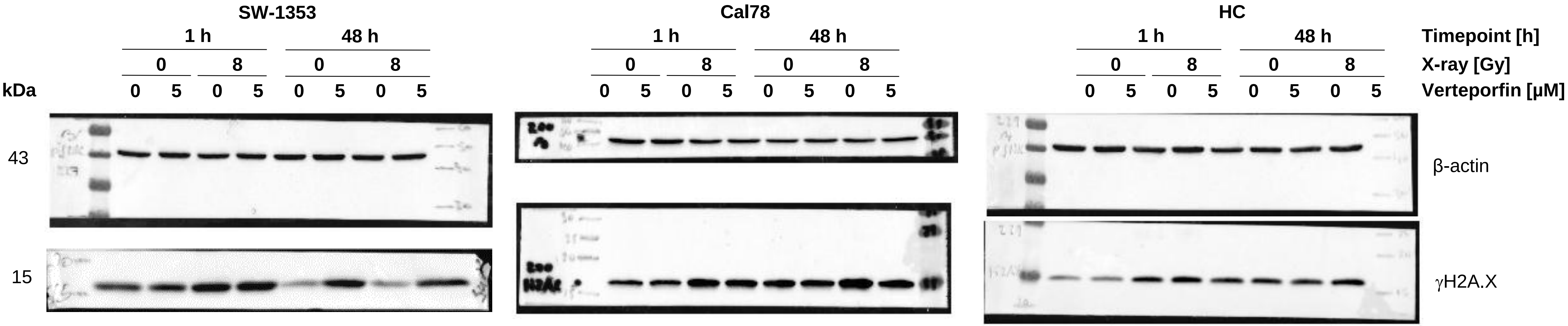

Supplement: Supplementary file 1 — Supplementary material [file mmc1.pdf]
